# Protective Effect of Crocin on Malathion-induced Cardiotoxicity in Rats: A Biochemical, Histopathological and Proteomics Study

**DOI:** 10.22037/ijpr.2020.111836.13385

**Published:** 2021

**Authors:** Soudabeh Balarastaghi, Rezvan Yazdian-Robati, Faezeh Vahdati Hasani, Hossein Hosseinzadeh, Khalil Abnous, Mohsen Imenshahidi, Leila Mohammadzadeh, Ruth Birner-Gruenberger, Bibi Marjan Razavi

**Affiliations:** a *Department of Pharmacodynamy and Toxicology, School of Pharmacy, Mashhad University of Medical Sciences, Mashhad, Iran. *; b *Molecular and Cell Biology Research Center, Faculty of Medicine, Mazandaran University of Medical Sciences, Sari, Iran. *; c *Pharmaceutical Research Center, Pharmaceutical Technology Institute, Mashhad University of Medical Sciences, Mashhad, Iran. *; d *Department of Medicinal Chemistry, School of Pharmacy, Mashhad University of Medical Sciences, Mashhad, Iran.*; e *Food and Drug Control Laboratory, Food and Drug Vice Presidency, Mashhad University of Medical Sciences, Mashhad, Iran. *; f *Institute of Pathology, Research Unit Functional Proteomics and Metabolic Pathways, Medical University of Graz, Graz, Austria. *; g *Targeted Delivery Research Center, Pharmaceutical Technology Institute, Mashhad University of Medical Sciences, Mashhad, Iran. *

**Keywords:** Malathion, Crocin, Cardiotoxicity, Proteomics, Oxidative stress

## Abstract

In this study, the protective effect of crocin on malathion (MTN) induced cardiotoxicity in rats in subacute exposure was evaluated. Rats were divided into 6 groups; control (normal saline); MTN (100 mg/kg); MTN + crocin (10, 20 and 40 mg/kg) and MTN + vitamin E 200 IU/kg. Treatments were continued for two weeks. Creatine phosphokinase MB (CK-MB), malondialdehyde (MDA) and glutathione (GSH) levels were evaluated in heart tissue at the end of treatments. The effect of crocin and MTN on histopathological changes in rat cardiac tissue was also investigated. The alteration of protein profile in the heart of the animals exposed to MTN was evaluated by proteomic approach through two-dimensional gel electrophoresis followed by matrix-assisted laser desorption/ionization-time of flight (MALDI-TOF) software. MTN induced histopathological damages and elevated the level of cardiac marker CK-MB (*P < *0.01). The level of MDA increased and the level of GSH reduced (*P < *0.001). MDA levels were reduced in all crocin plus MTN groups (*P < *0.001) and vitamin E plus MTN (*P* < 0.001) groups as compared to MTN groups. However, in the crocin (10 mg/kg) + MTN group, the content of GSH compared to MTN treated rats increased (*P < *0.001). Protein abundance analysis identified proteins implicated in cardiac necrosis, tricarboxylic acid cycle, cellular energy homeostasis, arrhythmias, heart development, heart failure and cardiovascular homeostasis to be affected by MTN. In summary, MTN may induce damage in the heart tissue of rats following subacute exposure and crocin, as an antioxidant, showed protective effects against MTN cardiotoxicity.

## Introduction

Organophosphate pesticides (OP), being cholinesterase inhibitors, are extensively used to control domestic and farming pests globally, especially developing countries (1). The common use of OP pesticides by community agricultural and health programs has led to severe environmental pollution that constitutes a potential health hazard by chronic or acute poisoning of humans (2). The residual amounts of these pesticides were detected in various agents like soil, water, fruits, vegetables, and other food products (3). 

The most important mechanism of OP poisoning is acetylcholine accumulation as a result of AChE inhibition (4). The induction of oxidative stress in animals and humans has been observed following exposure to OPs, which leads to the free radicals generation and then adaptive changes in the antioxidant defense system. The increased formation of reactive oxygen species (ROS) has been approved in the pathology of cardiovascular disease, dementia, cancer and metabolic disorders (5, 6). It has been proved that oxidative stress is considered one of the most important toxicity mechanisms induced by OPs in long-term exposures (7). Malathion (MTN) [O, O-dimethyl-S- (1,2-dicarcethoxyethyl) phosphorodithioate] is one of the most common OP pesticides which has been found in household and agricultural products and is widely used for pest control (8). The widespread usage of this pesticide makes it particularly vital to detect any related health risks. Several acute and chronic MTN poisoning among pesticide workers and children following accidental exposure have been reported. It is well documented that environmental contamination with MTN causes adverse health effects in humans, animals and wildlife (9).

Malathion is metabolized to malaoxon in the body, and this compound is 61 times more toxic than malathion (10). Its harmful effects have been demonstrated in various organisms, including honeybees and aquatic organisms, causing broad-ranging deformities (11). Therefore it is assumed that MTN poses a major risk for many organisms, including humans (12). Several different mechanisms of toxicity of MTN have been proposed, including inhibition of acetylcholinesterase activity in target tissues, several organ system dysfunctions including liver, pancreas, and the reproductive system, as well as changes in lipid metabolism, such as the elevation of low-density lipoprotein (LDL) and triglyceride levels in the blood (2,13-15). Besides, oxidative stress has been suggested as an alternative molecular mechanism for MTN toxicity: MTN increased MDA levels, which is an important marker of oxidative stress and the end product of lipid peroxidation, and it decreased the level of reduced GSH in the rat cortex and hippocampus (16). Crocin (crocetin digentiobiose ester), a unique water-soluble carotenoid, is one of the pharmacologically active ingredients of saffron (*Crocus sativus*) (17, 18). Several animal and clinical studies have revealed beneficial effects of saffron extracts and crocin, such as anti-tumor, anti-nociceptive, aphrodisiac, antidepressant, anxiolytic, anti-Alzheimer, anti-diabetic maculopathy, *etc*. through antioxidant and anti-inflammatory mechanisms (19-27). 

The cardioprotective effects of saffron and its components such as crocetin and crocin have been reported in different studies due to the modulation of endogenous antioxidant enzymatic activities (17). Previously our group has reported the protective effects of crocin on diazinon and acrylamide toxicities (28, 29). According to several studies, crocin, the main coloring pigment of *Crocus sativus *(*C. sativus*)*, *is safe. Hosseinzadeh *et al. *(2010) evaluated biochemical, hematological and pathological effects of crocin (up to 3 g, p.o. and i.p. as well as 15–180 mg/kg, i.p.) in mice and rats. The results showed that crocin at pharmacological doses did not exhibit any toxicity in different organs (30).

Since many literature data reported that OPs elicit different severe acute and long-term adverse effects and as far as we know, the toxic effects of subacute exposure of malathion on the cardiac system are not clear (31-34). Thus, this study was designed to determine the possible toxic effects of MTN on a specific biochemical cardiac enzyme (CK-MB), the MDA and glutathione levels, and morphological changes of the rat heart. Moreover, the possible protective effects of crocin on MTN toxicity were investigated. A proteomics method using two-dimensional gel electrophoresis and MALDI-TOF/TOF was used to identify differentially expressed proteins in rat hearts caused by the subacute administration of MTN. This is the first proteomics study that investigates protein changes caused by administration of malathion in the heart tissue to the best of our knowledge.

## Experimental

*Chemicals*

MTN was prepared from Ariashimi co., Tehran, Iran, and vitamin E was purchased from OSVE Pharmaceutical Co., Tehran, Iran. 2-thiobarbituric acid (TBA), potassium chloride, n-butanol, phosphoric acid and MDA were obtained from Merck, Germany. Reduced GSH were purchased from Sigma Aldrich, USA. Stigmas of *C. sativus* L. from Novin Saffron (collected from Ghaen, Khorasan province, Iran) was obtained and analyzed in accordance to the ISO/TS 3632-2. Crocin was extracted and purified as defined by Hadizadeh and colleagues (35). 

*Animals and treatment*

Adult male Wistar rats (weight 220–250 g) were supplied by the animal center of the School of Pharmacy of Mashhad University of Medical Sciences. Rats were maintained at a temperature of 23 ± 1 °C and on a 12 h light/dark cycle with free access to water and food during the experiments. Animal experiments were done according to the Mashhad University of Medical Sciences Ethical Committee Acts (941030). Rats were divided into 6 groups of 6 rats each: control group (normal saline); MTN treated group (100 mg/kg); MTN + crocin 10 mg/kg/day treated group; MTN + crocin 20 mg/kg/day treated group; MTN + crocin 40 mg/kg/day treated group; MTN + vitamin E 200 IU/kg treated group. MTN and crocin were administered daily via intraperitoneal injection (IP) for two weeks. Normal saline was given in the same way to control groups. Vitamin E was administrated intraperitoneally three days a week (36).

*Biochemical evaluation*

After two weeks, rats were sacrificed and blood samples were collected. Serum was prepared for biochemical tests. Heart tissues were removed, washed in normal saline and flash-frozen at -80 °C_._

*Cholinesterase activity assay*

Acetylcholinesterase activity in serum was measured by the Ellman method. The rate of hydrolysis of acetylthiocholine iodide was measured at 405 nm using the release of the thiol compound which produces a color-forming compound upon reaction with 5, 5-dithiobis-(2-nitrobenzoic acid) (DTNB). The absorbance was recorded at 0.5 min time intervals for 2 min. Cholinesterase activity was calculated as follows: Cholinesterase activity (mU/ml serum, at 25 °C) = change in absorbance in 30 s × 23400 (37).

*Measurement of MDA in the heart tissue*

MDA levels were determined according to the method of Fernandez *et al.* (38). This method is based on the spectrophotometric measurement of the color developed by the reaction of MDA to thiobarbituric acid (TBA). Briefly, heart tissues were homogenized for 2 min at 4 °C in 1.15% potassium chloride (KCl) solution to provide a 10% W/V homogenate. Three milliliter of phosphoric acid (H3PO4) (1% W/V) and 1of TBA (0.6% W/V) were then added to 0.5of the homogenate and the mixture was incubated for 45 min in a boiling water bath. After cooling with cold water, n-butanol (4 mL) was added and then the mixture stirred for 1 min. After vortex, the mixture was centrifuged for 20 min at 3000 ×g. The upper layer was transferred to the new tube and absorption of this layer was measured by the spectrometry device at 532 nm. The amount of MDA is reported as nmol/g tissue.

*Measurement of GSH in the heart tissue*

GSH content was measured based on the method of Moron *et al.* (39). Hearts were homogenized in ice-cold phosphate-buffered saline (PBS, pH 7.4) to obtain a 10% (w/v) homogenate. The reduced glutathione content was measured using (5, 5’-dithiobis-(2-nitrobenzoic­­­acid) (DTNB), which produced a yellow-colored 5-thio-2-nitrobenzoic acid (TNB). Then 10% trichloroacetic acid (TCA) and the equal amount of sample were mixed and centrifuged at 3000 ×g for 5 min. 0.5of 0.04% DTNB reagent and 2PBS (0.1 M, pH 8.0) were added to 0.5of supernatants (0.1 M, pH 8.0). And the absorption of the yellow-colored TNB was measured by the spectrometry apparatus at 412 nm. Tissue GSH content is reported as nmol/g tissue 

*Measurement of creatine phosphokinase-MB (CK-MB)*

The CK-MB activity in serum was measured using commercial colorimetric kits (Biosystem, Spain) by the auto-analyzer (Tokyo-Boeki Prestige). 

*Histopathological evaluation*

For histopathological evaluation, all animals’ hearts were excised at the end of the experiment, fixed in 10% v/v neutral buffered formalin and placed in paraffin wax. Tissue parts were cut in 6 µm slices and stained with hematoxylin and eosin. Histopathological assessments of the prepared tissues were performed using Olympus light microscope (BH-2, Japan), and the photos were taken with a digital camera attached to the microscope (Olympus, C-7070). 

*Two-dimensional gel electrophoresis sample preparation*

*Protein extraction*

Frozen heart tissues (0.2 g) from each group (control and MTN 100 mg/kg treated groups) were homogenized in 1 mL cold lysis buffer containing 2 M thiourea, 6 M urea, 2% (w/v) CHAPS, 2% (w/v) DTT, 20% (w/v) ampholyte (pH 3–10, Bio-Rad) and Halt™ Protease Inhibitor Cocktail using Polytron Homogenizer (IKA®T10, Germany). Using the Bio-Rad Protein Assay Kit and based on the Protocol factory, the concentration of supernatants was measured as described before (40).

*Two-dimensional gel electrophoresis*

For isoelectric focusing (IEF), was dissolved in 300 μL of the rehydration buffer (6 M urea, 0.3% w/v DTT, 2 M thiourea, 4% w/v CHAPS and 20% (w/v) ampholyte (pH 3–10) containing 300 μg of total protein, loaded on to each 17 cm 3–10 nonlinear IPG strips from Bio-Rad, USA, and actively rehydrated for 12 h at 50 V (37). IEF was performed using Protean IEF Cell (Bio-Rad, USA) at 20 °C and the following program: voltage started at 250 V, then linearly increased to 8000 V and kept constant at this voltage for 11 h. The current limit was set at 50 μA/gel. Then, the IPG strips were equilibrated in 6 mL of reduction buffer containing 37.5 mM Tris-HCl (pH 8.8), 6 M urea, 20% glycerol, 2% SDS, and 2% DTT for 15 min. For alkylation, the strips were equilibrated in 6 mL of solution containing 37.5 mM Tris-HCl (pH8.8), 6M urea, 20% glycerol, 2% SDS, and 2.5% w/v iodoacetamide for 15 min. After that, the strips were placed on top of 12% SDS-PAGE gels for the second dimension with the separation done at 120 V for 5 h, and then at 150 V until the tracking dye reached the end of the gel (41). After electrophoresis, gels were stained with colloidal Coomassie for 12 h (42).

*Analysis of images in-gel digestion and mass spectrometry*

After the staining stage (3 biological replicates/group), the gels were scanned by Image scanner III (Epson, Japan). Then, to find the different proteins expressed differently, the software Image Master Platinum 6.0 software was used (GE Healthcare, USA). Protein spots with more than a 1.5 fold change and significantly different intensities (*P* < 0.05) in control and MTN treated groups were considered as differentially expressed and selected for excision and mass spectrometric identification (43). Protein spots on the 2-D gels were excised from gels, transferred into a 1.5 mL microtube containing 1% acetic acid and sent to the Centre of Excellence in Mass Spectrometry at the University of York, UK, for in-gel digestion and identification by MALDI-TOF/TOF mass spectrometry. Gel pieces were reduced with DTT and treated with iodoacetamide for S-carbamidomethylation. They were then washed twice with 50% (v/v) aqueous acetonitrile (ACN) containing 25 mM ammonium bicarbonate and once with 100% ACN. After removing ACN, gel slices were dried in a vacuum concentrator for 20 min and rehydrated with 10 μL of 0.02 μg/μL sequencing-grade modified porcine trypsin (Promega). After 5 min, enough 25 mM ammonium bicarbonate solution) was added to cover the gel pieces. Digests were incubated at 37 °C overnight. The next day, extracted peptide mixtures (1 μL) were applied directly onto the ground steel MALDI target plate, followed immediately by mixing with 1 μL of a freshly-prepared 5 mg/mL of 4-hydroxy-α-cyanocinnamic acid (Sigma) solution in 50% aqueous (v/v) ACN, 0.1% trifluoroacetic acid (v/v). The BrukerUltraflex III in reflectron mode, equipped with an Nd: YAG smart beam laser, was applied for obtaining positive-ion MALDI mass spectra. MS spectra were acquired over a mass range of m/z 800–5000 and externally calibrated against an adjacent spot containing six standard peptides (Angiotensin I, 1296.685; des-Arg1-Bradykinin, 904.681; Glu1-Fibrinopeptide B, 1750.677; ACTH (18–39 clip), 2465.198; ACTH (1-17clip), 2093.086; ACTH (7–38 clip), 3657.929). For MS/MS fragmentation, the ten strongest peaks of interest with an S/N greater than 30 were chosen. Fragmentation was done in LIFT mode without the introduction of a collision gas. Spectral processing and peak list generation for both the MS and MS/MS spectra were performed by Brukerflex Analysis software (version 3.3).

Tandem mass spectral data were submitted to database search using a locally-running copy of the Mascot software (Matrix Science Ltd., version 2.5) through the Bruker BioTools interface (version 3.2). Search criteria included Enzyme Trypsin; Fixed modifications, Carbamidomethyl (C); Variable modifications, Oxidation (M); Peptide tolerance, 100 ppm; MS/MS tolerance, 0.5 Da; Instrument, MALDI-TOF/TOF (42).

*Classification of proteins*

The PANTHER online database (http://www.pantherdb.org) was utilized for functional classification of significantly altered proteins. PANTHER is a library of protein families and Subfamilies that demonstrate the biological functions, protein classes, and pathways (44).

*Statistical analysis*

Statistical analysis of MDA, GSH, and CK-MB aswas done by One-way Analysis of Variance (ANOVA) followed by Tukey post-hoc test using Graph Pad Prism version 6.00 for Windows, GraphPad Software. Protein intensities, expressed as a percent of total gel staining, were analyzed using Student’s *t*-test (GraphPad Prism version 6.00). All data were reported as mean ± standard error of the mean (SEM). *P*-values less than 0.05 were considered statistically significant.

## Results

*Biochemical evaluation*

**Figure 1:**
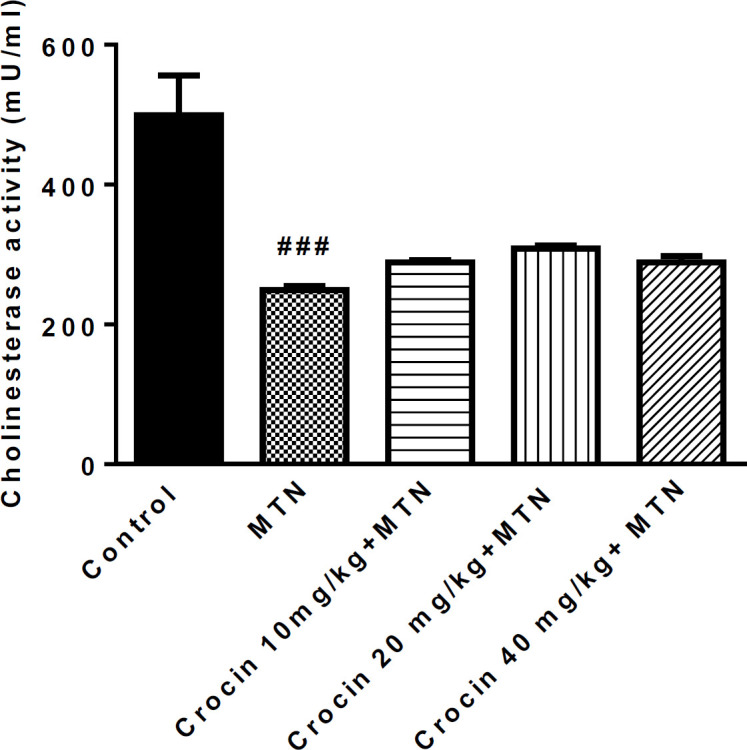
Effect of malathion and crocin treatment (2 weeks) on AChE activity. Data are presented as mean ± SEM. ^# # #^*P* < 0.001 compared to control. Tukey Kramer test, n = 6, MTN (malathion)

A significant reduction was observed in serum cholinesterase activity in the MTN group compared to the control group (499.3 ± 23.2 *vs*. 249 ± 3.005) (*P <* 0.001), but there was no significant difference between MTN plus crocin and MTN groups (Figure 1). 

**Figure 2:**
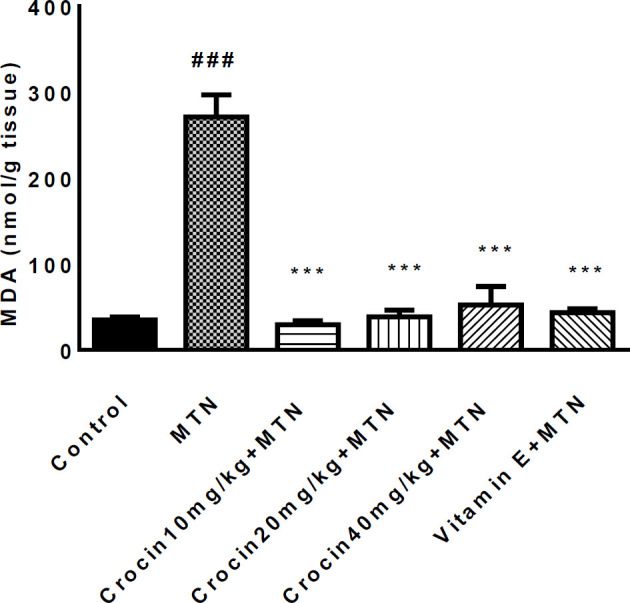
Effect of malathion and crocin treatment (2 weeks) on MDA Level in the heart rat tissues. Data are shown as mean ± SEM, ^*** ^*P* < 0.001compared to malathion group, ^###^*P* < 0.001compared to control group. Tukey–Kramer test, n = 6

**Figure 3:**
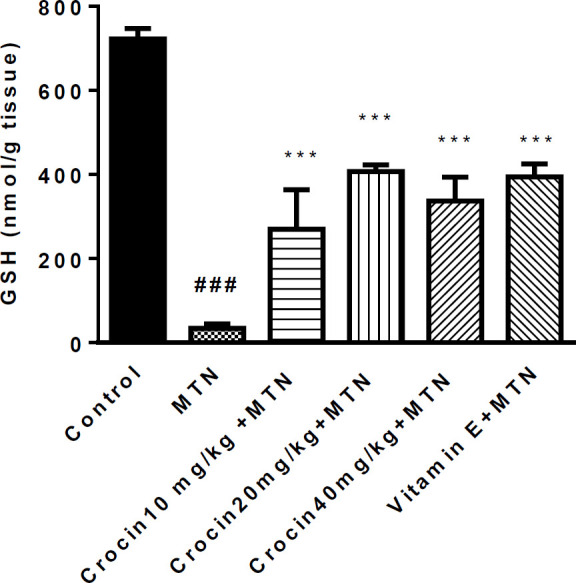
Effects of malathion and crocin treatment (2 weeks) on GSH level in the heart rat tissues. Data are shown as mean ± SEM, ^*** ^*P* < 0.001compared to malathion group, ^###^ *P* < 0.001 compared to control group. Tukey–Kramer test, n = 6

**Figure 4:**
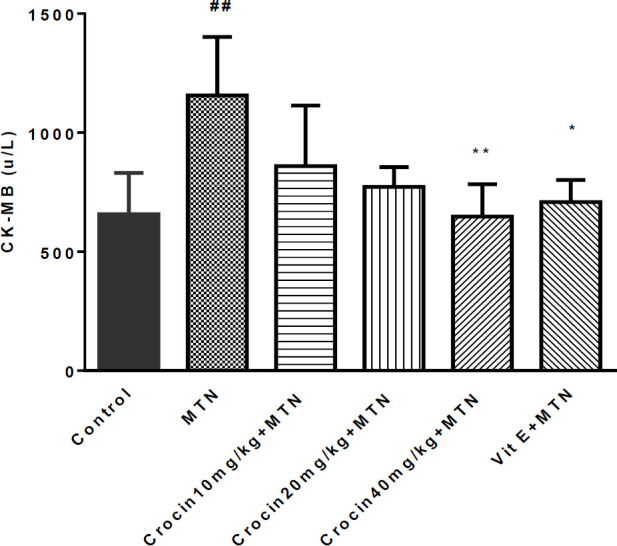
Effects of malathion and crocin treatment (2 weeks) on CK-MB activity. Data are shown as mean ± SEM, ^**^P < 0.01 and ^*^P < 0.05 compared to malathion group, ^##^*P* < 0.01 compared to control group. Tukey–Kramer test, n = 6

A significant increase was detected in MDA level in the MTN treated group in comparison with the control group (271.2 ± 11.51 *vs.* 35 ± 1.59) (*P < *0.001) (Figure 2). MDA levels were significantly reduced in crocin 10 mg/kg plus MTN (29.44 ± 2.11), crocin 20 mg/kg plus MTN (38.82 ± 3.5), crocin 40 mg/kg plus MTN (52.5 ± 9.56) (*P < *0.001) and vitamin E plus MTN (43.5 ± 2.02) (P < 0.001). In MTN treated rats, cardiac content of GSH was significantly decreased compared to control (722 ± 11.07 *vs. *34 ± 4.63) (*P < *0.001, Figure 3). However, in crocin 10 mg/kg + MTN (269 ± 41.92), crocin 20 mg/kg + MTN (407 ± 7.24) and crocin 40 mg/kg + MTN (337 ± 25.2) the content of GSH compared to MTN treated rats significantly increased (*P < *0.001). Serum CK-MB activity showed high cardiotoxicity in MTN treated group (1156.85 ± 122.8 *vs.* 657.15 ± 43 ± 9.6) (*P < *0.01, Figure 4). Co-administration of 40 mg/kg of crocin reduced serum CK-MB (647.4 ± 60.92) activity as compared with the MTN group (*P < *0.01).

*Histopathological analysis*

**Figure 5:**
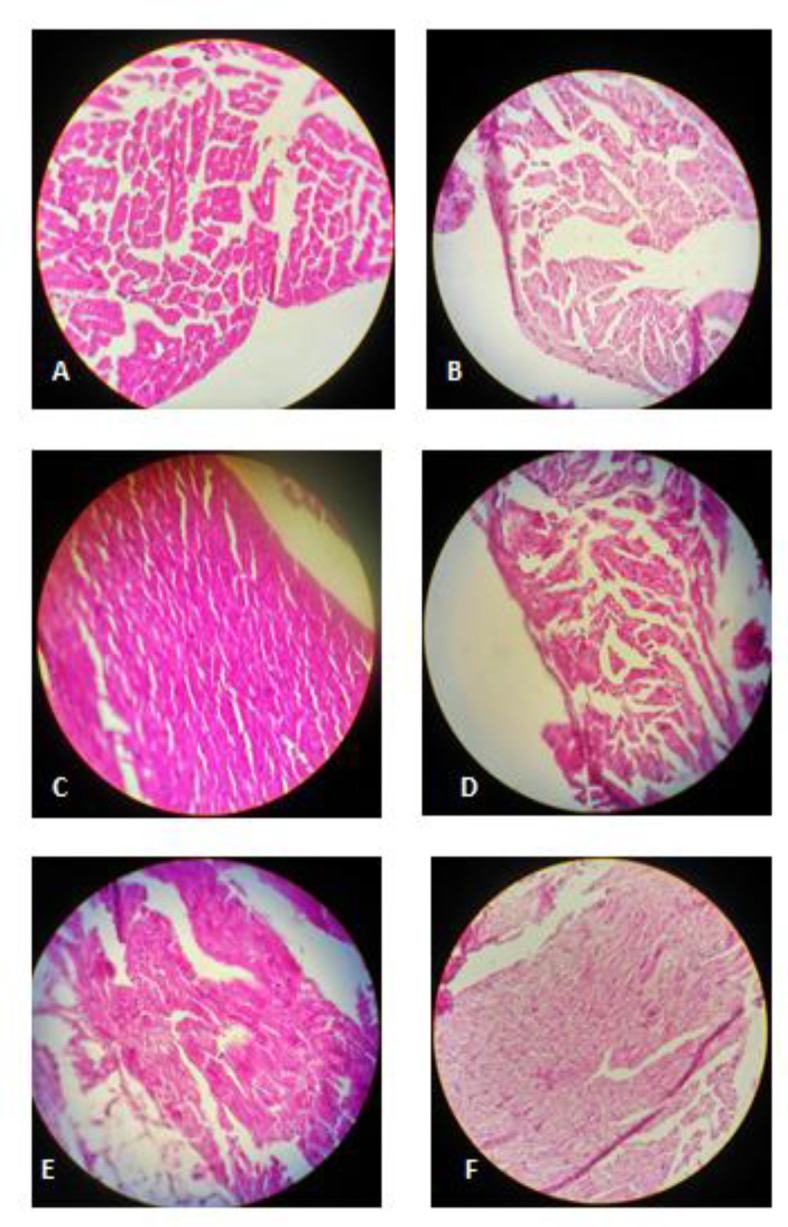
(A) Control rats: normal cardiac muscle cells, hematoxylin and eosin,× 400. (B) Malathion-treated rats: mild focal inflammation, myocytolysis and granular degeneration, hematoxylin and eosin,× 400. (C) Crocin 10 mg/kg+ malathion treated rats: normal cardiac muscle cells, hematoxylin and eosin,× 400. (D) Crocin 20 mg/kg+ malathion treated rats: myocytolysis, hematoxylin and eosin,× 400. (E) Crocin 40 mg/kg + malathion treated rats: mild focal inflammation, hematoxylin and eosin,× 400. (F) Vitamin E+ malathion treated rats: myocytolysis, hematoxylin and eosin, × 400

Histopathological changes of cardiac tissues are shown in Figure 5. Histological findings were normal in the control group (Figure 5A). Histological changes were observed in MTN treated group (Figure 5B) as mild focal inflammation, myocytolysis and granular degeneration. The cardiac lesions were significantly improved in the MTN plus crocin (10, 20 and 40 mg/kg) or vitamin E compared to the MTN-treated group (Figure 5). 

*Proteomics*

**Figure 6:**
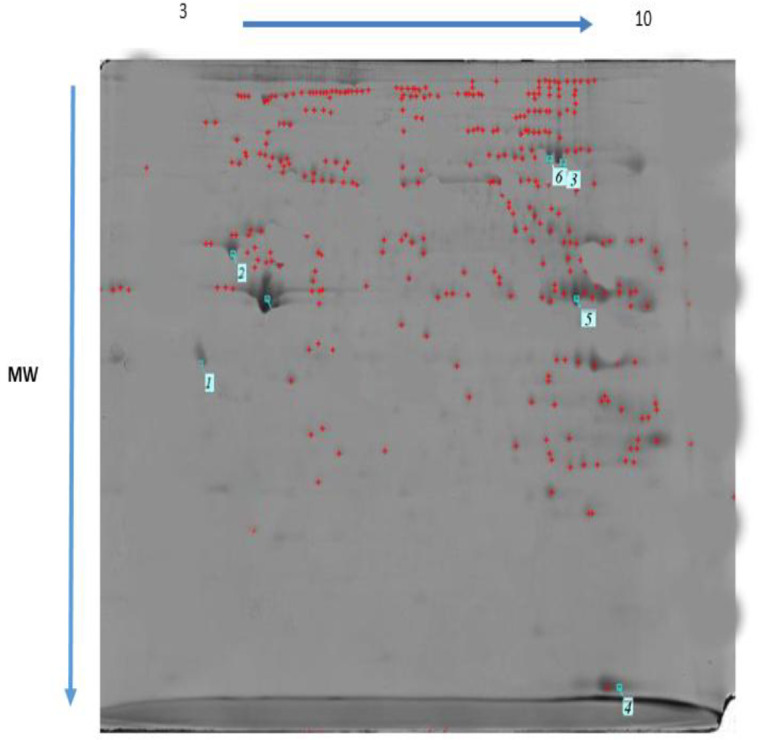
Two-dimensional gel electrophoresis of malathion treated rat heart (1 = Tropomyosin, 2 = ATP synthase, 3 = Desmin, 4 = L- lactate dehydrogenase, 5 = Malate dehydrogenase, 6 = Aconitate hydratase)

**Figure 7:**
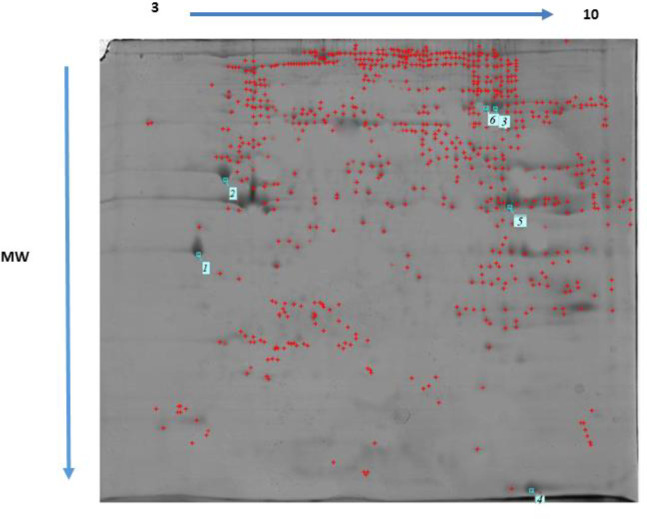
Two-dimensional gel electrophoresis of untreated rat heart

Differentially expressed proteins regulated by MTN exposure (100 mg/kg) and control in the rat heart tissue were identified and analyzed by 2-DE and MALDI TOF/TOF. Three rats from each group were randomly selected and subjected to 2-DE analysis. Representative examples of the 2-DE gel images of the proteins of the heart from MTN exposed rats are reported in Figures 6 and 7. 

MTN-therapyinduced changes of the protein and expression pattern in the heart proteome. Statistical analysis of differential protein expression revealed that seven total protein spots were differentially regulated between control and MTN-treated groups. Analysis of the total protein expression profile showed that 6 proteins were down-regulated while only one of them increased in the MTN-treated livers compared to the control samples. Table 1 provides more information about the differentially expressed proteins exposure to MTN, including SwissProt accession number, fold-change, MASCOT protein score, matched peptide sequenced, expectation-value and a brief description the biological function or molecular pathways. 

*Pathway analysis*

**Figure 8:**
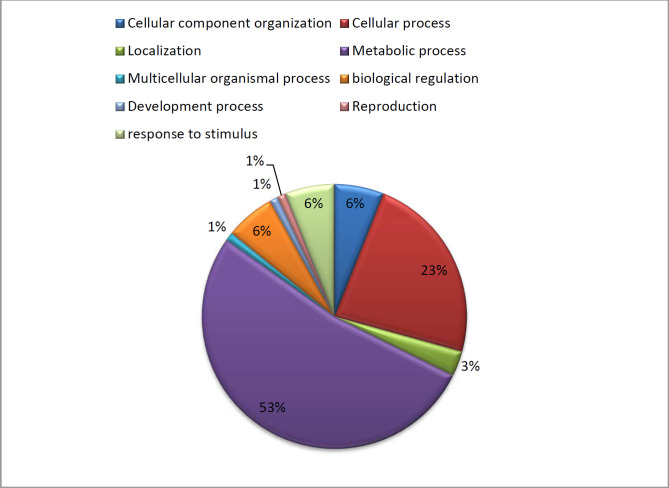
Functional classification of the differentially expressed proteins in the heart regulated by MTN exposure according to biological processes using the PANTHER classification system. Numbers indicate the percentage of protein against the total number of proteins

Using the PANTHER database (http://panther.appliedbiosystems.com/), the molecular function, biological process and signaling pathway of each identified protein was investigated. Differentially expressed proteins were classified in 9 pathways including; localization 3%, cellular process 23%, reproduction 1%, cellular component organization 6%, biological regulation 6%, developmental process 1%, metabolic process 53% and response to stimulus 6% (Figure 8). 

**Table 1:** Differentially expressed protein species in the heart of rats exposed to malathion as identified by MALDI-TOF/TOF and MASCOT software

**Protein name**	**Swiss Prot accession number**	**Fold-change (MTN/control)**	**Protein score**	**%sequence coverage**	**Matched peptides**	**Expectation-value**	**Matched peptide sequenced**	**Biological function or Molecular ** **‎** **pathways ** **‎** **(PANTHER ** **‎** **classification** **‎‎** **)** **‎**
Calreticulin	P18418	0.60	92	6	2(2)	0.0012	K.EQFLDGDAWTNR.W	Metabolic process
Tropomyosin	P58775	0.24	754	29%	9(9)	1e-10	K.KATDAEADVASLNR.R	Cellular component organization or biogenesis, cellular process, developmental process, metabolic process, multicellular organismal process
L-lactate dehydrogenase	P42123	1.57	513	19%	7(7)	8e-11	K.IVADKDYSVTANSK.I	Metabolic process
ATP synthase	P15999	0.56	1004	25%	10(10)	5.6e-11	R.EVAAFAQFGSDLDAATQQLLSR.G	ATP synthesis
Malate dehydrogenase	O88989	0.26	549	22%	7(7)	7.1e-12	K.ELTEEKETAFEFLSSA.-	Metabolic process
Aconitate hydratase, mitochondrial	Q9ER34	0.6	847	15%	10(10)	2.5e-10	K.FKLEAPDADELPR.S	Metabolic process ,TCA cycle
Desmin	P48675	0.56	751	21%	9(9)	3e-11	R.FASEASGYQDNIAR.L	developmental process cellular component organization or biogenesis

## Discussion

MTN is one of the most broadly used organophosphate compounds applied in agriculture as a pesticide, as an ectoparasiticide in veterinary practice, control of public health programs, and food preparation and processing areas (45, 46). In the present study, MTN (100 mg/kg) exhibited cardiac toxicity in subacute exposure (14 days) by inducing morphological damages and elevating the cardiac marker CK-MB level in serum. It has been shown that chronic exposure to OPs introduces oxidative stress damage and free radical production in different organs, such as the cardiovascular system (28, 47 and 48). Oxidative stress in cardiac tissues appeared to be responsible for these morphological and biochemical changes since co-administration with crocin in all doses of vitamin E was protective against MTN-induced cardiac toxicity by decreasing oxidative stress. Several studies reported that subacute exposure to Ops, such as MTN, inhibited cholinesterase enzyme activity and caused oxidative stress. Moreover, MTN was shown to produce free radicals in different organs, such as the cardiovascular system (29, 49-51). Our data revealed that the cardiac level of MDA was significantly enhanced after exposure to MTN. MDA as the common product of lipid peroxidation is widely used to assess the presence of free radicals and lipid peroxidation in different toxicity and disease studies, including in cardiovascular toxicity (52-54). Another biochemical marker we also measured in this work was GSH. GSH is an antioxidant marker, significantly reduced in MTN-treated group compared to the control group. In the present study, vitamin E or α-tocopherol, the biologically most active antioxidants, were utilized as positive controls. Our histopathological findings indicated that sub-chronic exposure to MTN could induce congestion, infiltration of inflammatory cells and multifocal necrosis in cardiac tissue. Crocin as a potent antioxidant agent showed cardioprotective effects (29, 55). Co-administration of Crocin and MTN as compared to MTN alone decreased the cardiac MDA level and the serum CK-MB concentration and increased the GSH content in the heart tissue. Crocin also protected the heart from histological changes induced by MTN, probably via its antioxidative and anti-inflammatory properties (56, 57). The protective effect of crocin may also be related to its inhibitory effect on lipid peroxidation leading to stabilization of membranes thereby blocking the release of cardiac enzymes (56). A proteomics approach was employed in the present study to identify if protein expression was changed in response to subacute exposure to MTN (Table 1). Seven proteins were found to be differentially regulated following MTN exposure compared to the control group. Our results confirmed a good agreement between well-known biomarkers of human cardiac disease and cellular response to MTN. Besides, using the proteomic approach, we revealed that mitochondrial proteins showing differential expression in response to MTN. ATP synthase, aconitate hydratase, malate dehydrogenase, and L-lactate dehydrogenase (LDH) as mitochondrial enzymes changed significantly in the MTN-exposed group. Involvement of mitochondrial injuries following exposure to OPs has been reported by many researchers (27, 58). Multiple lines of evidence have demonstrated that OPs could alter mitochondrial respiration and respiratory chain enzyme activity (59, 60). Mitochondria have a vital role in ATP production and the ROS, so dysfunction of mitochondria in the OPs toxicity involved in multisystem disorders in different organs including liver, heart and neurons (61, 62). Indeed, changes in the levels of these mitochondrial enzymes may the most relevant signal of heart toxicity of MTN (36). LDH, as an important glycolytic enzyme, is a potential biomarker in toxicology and clinical chemistry if detected extracellularly as a sign of cell, tissue and organ damage (63). Previously, it has been demonstrated that the LDH activity altered after exposure to toxic compounds or oxygen stress (64-67). Venkatesan *et al.* have also reported that malathion treatment enhanced LDH release in a dose-dependent manner and induced neurotoxicity in N2a neuroblastoma cells (68). Elevated LDH also entirely consistent with that observed by Kalender *et al.* (69). Increased LDH in the malathion-treated group in the present study may indicate toxic effects of MTN in the heart. Multiple studies have reported an association between ATP synthase and exposure to the OPs pesticide (31, 33 and 34). Decreases in ATP synthase level in OPs treated groups compared to control supported by several studies (70, 71). Acute exposure to MTN in a rat model induced cardiac failure, diminished ATP production and affected ADP/ATP ratio in heart tissue cell line (32). Decreased ATP synthase as observed in response to MTN may reflect the inability of the cells to maintain a normal energy level (72). Down-regulation of aconitate hydratase suggesting overproduction of superoxide anion and hydrogen peroxide in rat heart mitochondria in subacute intoxication with MTN (58).

Upon exposure to MTN, the level of malate dehydrogenase- a key enzyme in the Krebs cycle- in rat heart tissue was significantly reduced. In the present study, this reduction in malate dehydrogenase indicates a decrease in the respiration rate due to toxicity induced by MTN (73). Decreased level of malate dehydrogenase results in lesser production of ATP in the mitochondria which may prevent numerous important metabolic functions in the animals (74). Calreticulin, a Ca^2+^-binding chaperone in the endoplasmic reticulum (ER), is a regulator of calcium homeostasis through modulation of ER Ca^2+^ storage and transport (75). Furthermore, calreticulin affects many aspects of cellular processes, including cardiac development, muscle contraction, cell adhesion, adipocyte differentiation, innate immunity, steroid-sensitive gene expression, apoptosis, and stress responses (76, 77). It is possible that decreased calreticulin in the heart tissue following MTN treatment could be responsible for lipid production, previously proposed (69). In our proteomic study, reduced-expression of calreticulin was detected after exposure to MTN. Tropomyosin and desmin were found to decrease in the heart after MTN administration. Tropomyosins are actin filament binding proteins, possessing significant roles in regulating cardiovascular homeostasis and modulation of endothelial cell function (78). Desmin, as a muscle-specific protein and a crucial intermediate filament in the heart, involves inhibition of cardiac conduction, cell architecture, arrhythmias, mitochondrial function and behavior and restrictive heart failure (70, 79). It has been reported a heart without desmin is characterized by cardiomyocyte degeneration and a dilated cardiomyopathy (71).

## Conclusion

In conclusion, our data indicate that crocin has protective effects against MTN-induced cardiotoxicity through attenuating lipid peroxidation and increasing GSH content. Protein analysis identified proteins implicated in cardiac necrosis, tricarboxylic acid cycle, cellular energy homeostasis, arrhythmias, heart development, heart failure and cardiovascular homeostasis to be affected by MTN. 
